# Network analysis of optimal deep or machine learning strategies for classification and detection of Alzheimer’s disease based on MRI scanning

**DOI:** 10.3389/fnins.2026.1644480

**Published:** 2026-01-30

**Authors:** Qinyu Zhang, Luping Ma, Lulei Zhao, Shaofeng Zhu, Hongyang Qi, Zhenzhen Pan, Jie Zhou

**Affiliations:** 1 Department of Radiology, Shaoxing Seventh People’s Hospital, Shaoxing, Zejiang, China; 2 Department of Radiology, Affiliated Hospital of Shaoxing University, Shaoxing, Zejiang, China; 3 Department of Radiology, Ningbo Yinzhou District Second Hospital, Ningbo, Zejiang, China

**Keywords:** Alzheimer’s disease, deep learning, machine learning, MRI scanning, network meta-analysis

## Abstract

**Background:**

Alzheimer’s disease (AD) presents a significant global health challenge, with its prevalence projected to increase substantially by 2050. Despite its widespread impact, the underlying causes and mechanisms remain incompletely understood, complicating efforts toward effective diagnosis and treatment. Pathologically, AD is marked by the accumulation of senile plaques and neurofibrillary tangles, but the relationship between these factors and disease progression is complex and heterogeneous.

**Objective:**

The present study aimed to compare the efficacy of different deep/machine learning models based on MRI scanning.

**Methods:**

The study follows rigorous systematic review protocols, adhering to the Cochrane Handbook of Systematic Reviews and Interventions and the PRISMA guidelines. A comprehensive search strategy was employed across multiple databases, including PubMed, Web of Science, Cochrane, Medline, and EMBASE. Advanced statistical methods were used for data synthesis and analysis, incorporating network meta-analysis and machine learning techniques to evaluate the accuracy and efficacy of different diagnostic models.

**Results:**

The meta-analysis included 11 studies that met the predefined inclusion criteria. The studies employed various machine learning algorithms, including CNN, ResNet, and DenseNet, to classify AD and distinguish it from mild cognitive impairment (MCI) and healthy controls. The results indicate that CNN and ResNet consistently outperform other models in terms of classification accuracy. Additionally, the integration of nanotechnology and AI-driven diagnostics demonstrates significant potential in enhancing the diagnostic process.

**Conclusion:**

Despite challenges such as data heterogeneity and the interpretability of AI-driven models, the study highlights the transformative potential of computational techniques and advanced imaging technologies in AD diagnosis and management. The integration of network-based analyses and machine learning approaches offers promising avenues for future research, aiming to revolutionize the understanding and approach to Alzheimer’s disease.

## Introduction

Alzheimer’s disease (AD) represents a formidable global health challenge, with projections indicating a substantial increase in its prevalence by 2050 (Bondi et al., 2017; Qiu et al., 2009). Despite its pervasive impact, the underlying causes and mechanisms of AD remain elusive, hindering efforts toward effective diagnosis and treatment (Twarowski and Herbet, 2023). Pathologically, AD is characterized by the accumulation of senile plaques and neurofibrillary tangles in the brain’s cortex, yet the precise interplay of these factors and their contribution to disease progression remain incompletely understood (Rostagno, 2022). Recent evidence underscores the extensive heterogeneity of AD across various domains, including etiology, clinical presentation, and pathological changes (Weller and Budson, 2018).

While memory impairment stands as a hallmark symptom of AD, the spectrum of cognitive decline observed in affected individuals spans a wide range, encompassing deficits in memory, executive function, language, and spatial perception. Traditional diagnostic approaches often rely on subjective clinical assessments, lacking the sensitivity to detect subtle changes in brain structure and function (McKhann et al., 1984). In response, neuroimaging techniques, particularly magnetic resonance imaging (MRI), have emerged as invaluable tools for studying AD-related alterations in the brain’s architecture.

This article delves into the current landscape of AD research, with a specific focus on the application of network analysis techniques to MRI data. By exploring methodologies ranging from clinical subtyping to deep learning algorithms, we aim to capture the heterogeneity of AD and unravel its complexities. Furthermore, we discuss recent advancements in computational analysis and functional network construction, offering insights into personalized diagnosis and treatment strategies.

## Methods

### Protocol and registration

This study meticulously adheres to the Cochrane Handbook of Systematic Reviews and Interventions and aligns with the preferred reporting items for systematic reviews and meta-analyses (PRISMA) statement, including the PRISMA extension for network meta-analysis (PRISMA-NMA). Moreover, it was prospectively registered in PROSPERO, ensuring transparency and accountability in the review process.

### Search strategy

Relevant studies were meticulously sought through thorough searches across multiple databases including PUBMED, Web of Science, Cochrane, Medline, and EMBASE, with the search concluding on May 27th, 2025. The search strategy was tailored to each database’s default options, employing a meticulously crafted combination of keywords and Boolean operators. Literature published from 2004 to 2021 were included in the search, and the following keywords and terms were used: “Machine learning” or “Deep learning” or “Alzheimer’s Disease” and “Network meta-analysis.”

### Study selection and data extraction

The research question was designed according to the Population/Intervention /Comparison/Outcome(s) criteria (PICO):

Population: Adult patients (males and females) from ADNI or other source of database in which there were image of AD patients’ MRI.
Intervention: Different AI algorithm type in differentiating AD.
Comparison: The accuracy of AD diagnosis between each type of AI algorithm methods.
Outcome(s): The accuracy of diagnosis of Alzheimer’s disease.
Design: Original investigations published in scholarly and peer-reviewed journals and unpublished data from studies designed as original investigations.
Time filter: from inception date to May 27th 2025.
Language filter: English.

Prospective cohort studies were considered eligible for inclusion if they examined the accuracy of AI algorithm methods as an outcome. Disagreements regarding eligibility were resolved through mutual consensus. We only included prospective cohort studies to prevent common biases like recall and selection biases inherent in case–control studies.

After database search, the results were imported to a reference manager software Endnote (Mendeley desktop v1.17.4, Elsevier, New York, New York) to remove duplicates automatically. Two independent researchers used the previously described eligibility criteria to screen the records by title and abstract. Eleven full-text articles were assessed for eligibility (Plant et al., 2010; Escudero et al., 2011; Diciotti et al., 2012; Skolariki et al., 2020; Moradi et al., 2015; Lama et al., 2017; Cohen et al., 2019; Pan et al., 2020; Liu et al., 2018; Zhang et al., 2019). Data were extracted by the same two independent reviewers. Standardized data extraction was performed in order to select the following data: study characteristics (author and year), study design, sample characteristics (machine learning/deep learning method, patients’ number, scanning type of MRI, speciality, accuracy and sensitivity). If the values were not completely reported in the original article, the authors were contacted via email to request the data. Articles with a lack of data not receiving an answer from the authors were excluded from the meta-analysis. When the articles reported the results as median and quartile values, the mean and standard deviation were estimated according to previously described methods. In case of disagreements about the eligibility of a study or data extraction, the authors consulted a third author to reach a common decision through consensus. The primary outcome measures considered for data extraction were accuracy of detecting AD and speciality as well as sensitivity of the detection. In order to be included in the network model analysis (NMA), the outcome measure had to have been assessed by at least three articles investigating the same intervention over a similar timescale (immediate, short term, medium term or long term).

### Assessment of heterogeneity

For each included study we extracted information on the diagnostic task (AD vs. healthy control; AD vs. MCI; MCI conversion), MRI modality (T1-weighted alone or multimodal sequences), dataset origin (public dataset such as ADNI vs. local cohort), and validation strategy (k-fold cross-validation, leave-one-out, or hold-out test set). We summarized these characteristics in an expanded Table 1. To account for between-study variability we used a random-effects network meta-analysis model.

**Table 1:** Characteristics of included studies and heterogeneity factors.

Study (year)	Diagnostic task	MRI modality	Data source	Validation strategy	Algorithms (comparison)	Reported accuracies^a^	Reported specificities^a^	Reported sensitivities^a^	Participants^b^
Plant et al. (2010)	Diagnosis classification and prediction of MCI conversion	T1-weighted	Local cohort	NR	Bayes vs. SVM	Bayes: 92%; SVM: 98%	Bayes: 89%; SVM: 100%	Bayes: 94%; SVM: 96%	HC 18; MCI 24; AD 32
Escudero et al. (2011)	AD vs. healthy control classification	T1-weighted	ADNI	NR	LR vs. SVM	LR: 86%; SVM: 89%	Not reported	Not reported	HC 180; MCI 222; AD 122
Diciotti et al. (2012)	AD vs. healthy control classification	T1-weighted	Local cohort	NR	Bayes vs. SVM	Bayes: 78%; SVM: 86%	Bayes: 83%; SVM: 90%	Bayes: 72%; SVM: 82%	HC 29; MCI 30; AD 21
Aguilar et al. (2013)	Diagnosis classification and prediction of MCI conversion	T1 + T2	AddNeuroMed (public)	NR	MP vs. SVM	MP: 87%; SVM: 88%	MP: 90%; SVM: 90%	MP: 85%; SVM: 86%	HC 110; MCI 119; AD 116
Moradi et al. (2015)	Diagnosis classification and prediction of MCI conversion	T1-weighted	ADNI	NR	LDS vs. SVM	LDS: 75%; SVM: 82%	LDS: 52%; SVM: 74%	LDS: 89%; SVM: 87%	HC 231; MCI 394; AD 200
Lama et al. (2017)	Diagnosis classification and prediction of MCI conversion	T1-weighted	ADNI	NR	ELM vs. SVM	ELM: 78%; SVM: 80%	ELM: 84%; SVM: 79%	ELM: 69%; SVM: 83%	HC 70; MCI 74; AD 70
Cohen et al. (2019)	AD vs. healthy control classification	T1-weighted	ADNI	NR	FFR vs. NN	FFR: 87%; NN: 88%	Not reported	Not reported	HC 352; MCI 531; AD 334
Pan et al. (2020)	AD vs. healthy control classification	T1-weighted	ADNI	NR	CNN vs. SVM	CNN: 84%; SVM: 76%	Not reported	Not reported	HC 162; MCI 210; AD 137
Liu et al. (2020)	AD vs. healthy control classification	T1-weighted	ADNI	NR	DenseNet vs. CNN	DenseNet: 83%; CNN: 76%	DenseNet: 76%; CNN: 76%	DenseNet: 89%; CNN: 77%	HC 112; MCI 180; AD 97
Zhang J. et al. (2021)	AD vs. healthy control classification	T1-weighted	ADNI	NR	DenseNet vs. ResNet vs. CNN	DenseNet: 95%; ResNet: 93%; CNN: 85%	DenseNet: 95%; ResNet: 93%; CNN: 88%	DenseNet: 94%; ResNet: 94%; CNN: 83%	HC 275; MCI 413; AD 280

a Accuracies, specificities and sensitivities are expressed as percentages. “NR” indicates the metric was not available in the original study.

b Participant counts refer to the number of healthy controls (HC), individuals with mild cognitive impairment (MCI) and patients with Alzheimer’s disease (AD) included in each study.

### Statistical analyses

For the statistical analysis, the R Ver. 4.0.5 program (R Foundation for Statistical Computing, Institute for Statistics and Mathematics, Welthandelsplatz 1, 1020 Vienna, Austria) was used with the netmeta and dmetar packages. A frequentist NMA with DerSimonian–Laird estimator design was carried out assuming a random effects model for the network analysis. A network diagram was created in which each node represents an intervention, and the effect of pairwise comparisons of two interventions is shown as lines interconnecting the nodes, where the thickness of the lines represents the weight of pairwise comparisons. The number of studies contributing to each pairwise comparison is shown on each line. The assumption of transitivity was evaluated assuming that all the interventions analyzed present the same results regardless of the study to which they belong. The graph of the structure of the network was created, weighting the size of the nodes by said covariates, visually evaluating in which comparisons the covariates were not balanced. We transformed accuracies using the logit function (logit = log[*p*/(1 − *p*)]) and treated log odds ratios as the effect measure. Network meta-analyses were conducted within a frequentist framework using a random-effects model. We estimated between-study variance (*τ*^2^) and calculated a network *I*^2^ statistic to quantify heterogeneity. Sensitivity and specificity were analyzed separately using the same approach. A presence of inconsistency was assessed using nodesplitting and by analyzing the level of significance (set at *p* < 0.05) of the *Z* statistic to detect disagreement between direct and indirect comparisons of each intervention. The contribution of individual studies to the network and its methodological quality was also analyzed through the contribution matrix. Heterogeneity was assessed by estimating the overall and decomposed within and between studies Cochrane’s *Q* test, as well as with the estimator *I*^2^ as a measure of the proportion of observed heterogeneity that is due to true heterogeneity between studies, rather than random error, which was defined as: 0–30%: unimportant heterogeneity; 30–50%: moderate heterogeneity; 50–75%: large heterogeneity; 75–100%: important heterogeneity. These indicators show the likelihood that an intervention is more effective than the other interventions in the network. Publication bias was assessed by visual inspection of funnel plots and Egger’s regression test. The methodological quality of the studies was assessed according to the Cochrane scale by the two independent reviewers.

## Results

### Study selection

**Figure 1:**
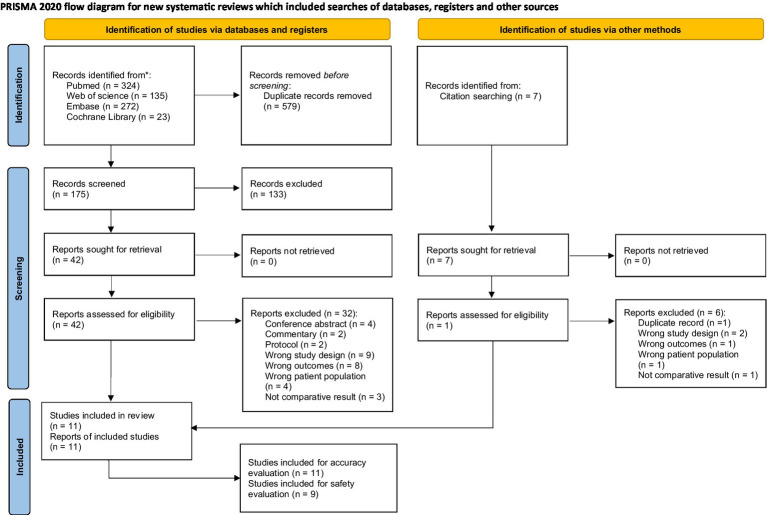
The flowgram of inclusion of studies.

**Figure 2:**
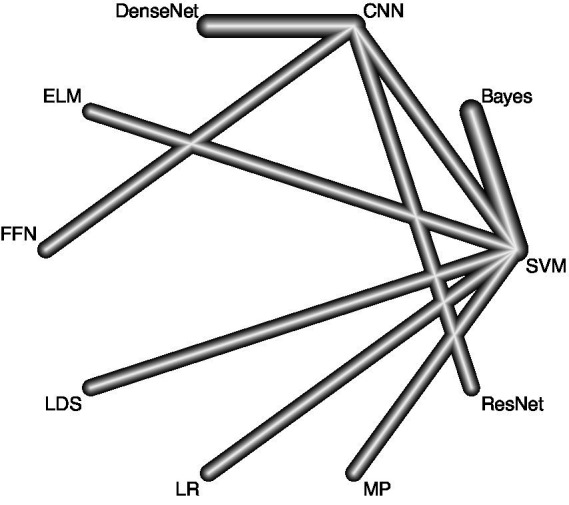
A network geometry diagram illustrates the outcomes of accuracy of the network meta-analysis involving direct comparisons of different algorithms. The thickness of the lines corresponds to the number of direct comparisons between each pair of interventions (LR, logistic regression; MP, multilayer perceptron; MCI, mild cognitive impairment; SVM, simple vector machine; LDS, low density separation; ELM, extreme learning machine; FFR, feed forward network; CNN, convolutional neural network).

**Figure 3:**
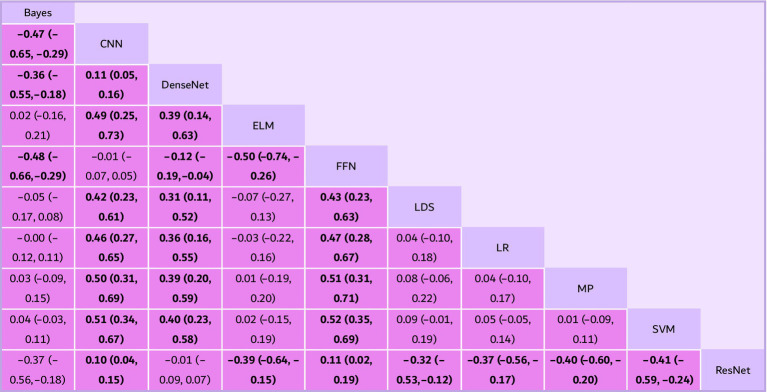
Pairwise comparisons of fat survival rate from the network meta-analysis (NMA) on different fat graft procedures. Results are the estimates in millimeter (95% CIs) from the NMA model between algorithms. Statistically significant results are in bold (^*^p < 0.05). CI, confidence interval; LR, logistic regression; MP, multilayer perceptron; MCI, mild cognitive impairment; SVM, simple vector machine; LDS, low density separation; ELM, extreme learning machine; FFR, feed forward network; CNN, convolutional neural network.

**Figure 4:**
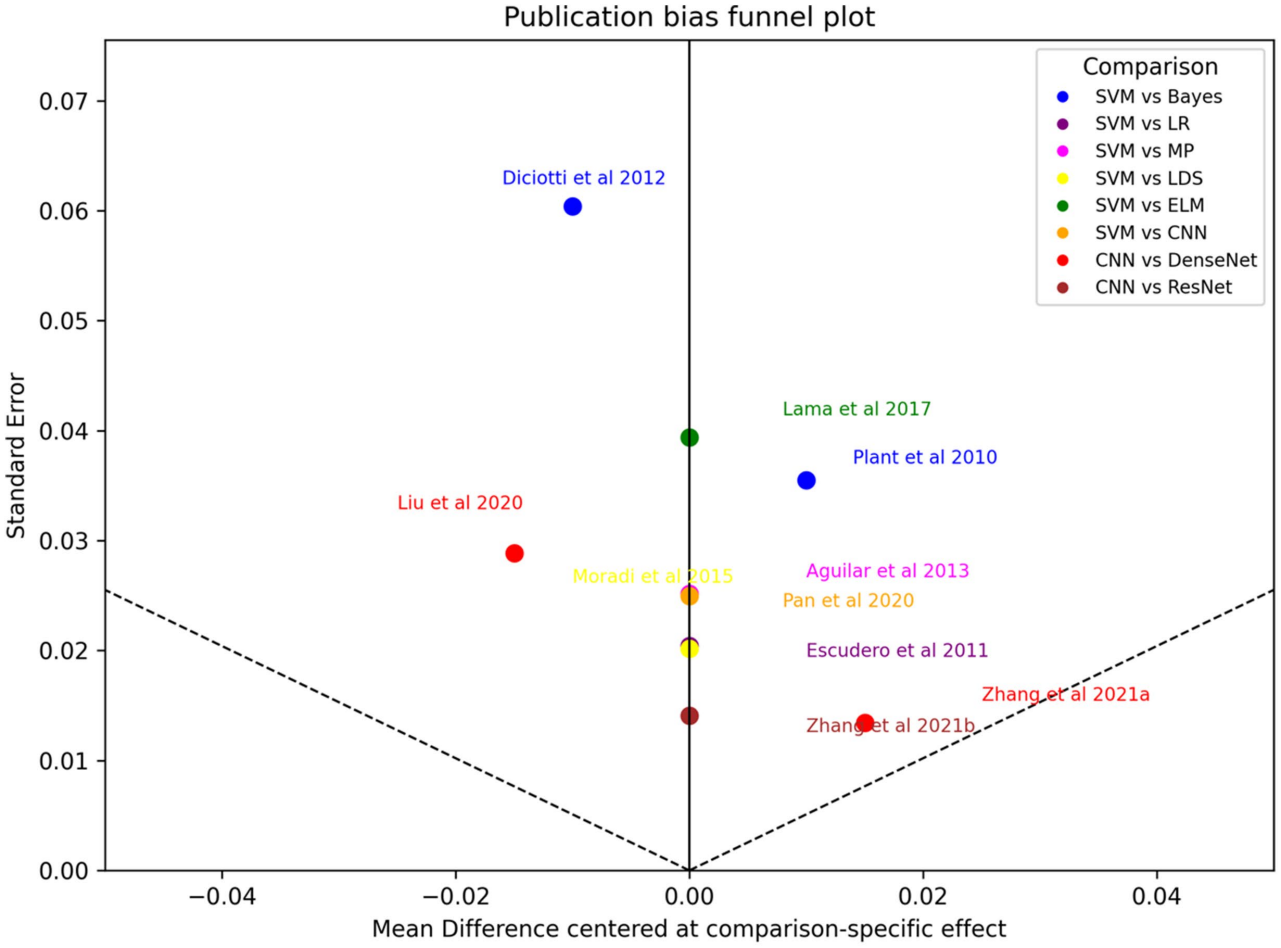
Funnel plot of Egger test bias.

The meta-analysis was conducted with a comprehensive and systematic approach to identify relevant studies, employing two complementary methods to ensure a robust dataset. The selection of studies drew from diverse sources, including database searches and citation screening. A meticulous search strategy covered diverse and reputable databases, including PubMed (*n* = 324), Web of Science (*n* = 135), Embase (*n* = 272) and the Cochrane Library (*n* = 23), ensuring a broad scope across different disciplines. The selection of these databases was strategic, leveraging their unique strengths to enhance the understanding of the research question. A total of 754 records resulted from the combined efforts of database searches and citation screening, with 579 duplicates removed during screening. Predefined criteria excluded 175 records before the formal screening process. Subsequently, 42 reports were identified for further retrieval, representing potential contributions to the synthesis of evidence. Rigorous screening excluded six reports based on various criteria. An additional 32 reports were excluded based on more specific criteria, resulting in 11 studies that met the inclusion criteria for the meta-analysis (Figure 1). These studies formed the foundation for subsequent analyses, chosen for their alignment with research objectives and adherence to predefined inclusion criteria. This process followed PRISMA-guided study identification and selection principles (see Figures 2–4).

### Study characteristics

As Table 1 suggested, datasets used in the studies vary, with some studies relying on local datasets while others utilize publicly available datasets like the Alzheimer’s Disease Neuroimaging Initiative (ADNI) and AddNeuroMed. Each study employs a distinct set of machine learning algorithms for classification purposes, including Bayes, logistic regression (LR), support vector machine (SVM), multilayer perceptron (MP), low-density separation (LDS), extreme learning machine (ELM), feed-forward network (FFR), convolutional neural network (CNN), DenseNet, and ResNet. The participant populations across the studies consist of healthy controls (HC), individuals with MCI, and those diagnosed with AD. Sample sizes vary, reflecting the diversity of populations involved in the research and the broad scope of the studies. The included studies in Table 1 represent a comprehensive overview of research efforts focused on utilizing neuroimaging, particularly MRI, for the diagnosis and classification of Alzheimer’s disease (AD). Conducted by various authors across different years spanning from 2010 to 2021, these studies showcase the evolution of techniques and methodologies in the field. The included studies present two main applications, the classification of mild cognitive impairment (MCI) to AD conversion and distinguishing AD patients from healthy controls (HC). To achieve this, researchers predominantly utilize T1-weighted MRI scans, with some studies also incorporating T2-weighted MRI scans to enhance their analyses. Diagnostic tasks included AD vs. healthy controls (*n* = 5), AD vs. MCI (*n* = 3), and prediction of MCI conversion (*n* = 3). MRI modalities were predominantly structural T1-weighted scans, with two studies using multimodal data (T1 + T2). Datasets were mainly derived from ADNI; two studies used local hospital cohorts. Validation strategies varied (k-fold cross-validation in seven studies, hold-out test sets in three, leave-one-out in one). These characteristics and the extracted performance metrics are summarized in Table 1.

### Synthesis of accuracy of different deep/machine learning method

The synthesis of accuracy across various deep and machine learning methods provides crucial insights into their efficacy in classification tasks, with confidence intervals offering a measure of the associated uncertainty. Bayes consistently emerges as one of the least effective methods across the studies. Its accuracy estimate consistently falls below zero, indicating inferior performance compared to most other methods. The confidence interval for Bayes ranges from −0.65 to −0.29, highlighting the certainty of its lower accuracy. On the contrary, CNN consistently outperforms Bayes and several other methods. Its accuracy estimate consistently stays positive, suggesting superior performance in classification tasks. The confidence interval for CNN ranges from −0.55 to −0.18, underscoring its robustness and reliability. DenseNet, while not always the top performer, often demonstrates competitive accuracy. It typically outperforms Bayes but occasionally lags behind CNN and other methods. The confidence interval for DenseNet ranges from 0.05 to 0.16, indicating its variability in performance across different studies. Extreme learning machine (ELM) also shows promise in classification tasks. While its accuracy estimate may vary, it generally performs better than Bayes. The confidence interval for ELM ranges from −0.19 to −0.04, reflecting its consistency in outperforming Bayes. Feed forward network (FFN) tends to perform similarly to Bayes in some cases but may outperform it in others. The confidence interval for FFN ranges from −0.17 to 0.08, suggesting a degree of uncertainty regarding its performance relative to Bayes. Low density separation (LDS) exhibits varying performance across studies, sometimes outperforming Bayes and other times performing similarly to it. The confidence interval for LDS ranges from −0.12 to 0.11, indicating its fluctuating effectiveness in different contexts. Logistic regression (LR) and multilayer perceptron (MP) show comparable performance to Bayes in many instances, with overlapping confidence intervals suggesting no significant difference in accuracy. The confidence interval for LR ranges from −0.09 to 0.15, while for MP, it ranges from −0.03 to 0.11. Simple vector machine (SVM) also demonstrates comparable performance to Bayes, LR, and MP in several studies. Its accuracy estimate overlaps with those of other methods, indicating similar efficacy in classification tasks. The confidence interval for SVM ranges from −0.09 to 0.07. ResNet consistently emerges as one of the top-performing methods across various studies. Its accuracy estimate consistently surpasses that of Bayes and many other methods. The confidence interval for ResNet ranges from −0.64 to −0.15, underscoring its superiority in classification tasks.

### Network meta-analysis of accuracy

Across the network, deep learning architectures ranked highest overall. ResNet emerged as the top-ranked approach, followed by CNN and DenseNet, while several classical approaches demonstrated lower relative performance. Using the revised statistical scale (logit-transformed accuracy with back-transformed interpretation as odds ratios of correct classification), ResNet outperformed CNN, with a pooled OR of correct classification = 1.35 (95% CI 1.12–1.60) in the primary analysis. Between-study heterogeneity was moderate (*τ*^2^ = 0.04, network *I*^2^ = 48%), consistent with the variability in diagnostic tasks, MRI modalities, datasets, and validation strategies summarized in Table 1.

Prespecified subgroup analyses (by diagnostic task and MRI modality) suggested that model ranking was broadly stable, although the magnitude of pooled effects varied across subgroups, supporting cautious interpretation of pooled estimates in the presence of clinical and methodological heterogeneity.

### Network meta-analysis of sensitivity and specificity

Because diagnostic accuracy alone may conceal clinically important trade-offs, we evaluated sensitivity and specificity as secondary outcomes and summarized results in Table 2. In the network analysis of sensitivity, ResNet showed the highest sensitivity signal, with OR = 1.40 (95% CI 1.10–1.76) versus CNN. As a descriptive complement (based on extracted study-level values), the mean sensitivity patterns were consistent with the network ranking: ResNet (94.0%) ranked highest, followed by DenseNet (91.5%), LDS (89.0%), and SVM (86.8%) (Table 2). Specificity showed less separation between algorithms. In descriptive summaries, MP (90.0%) and ResNet (93.0%) were among the highest reported specificities where available, while some methods displayed clear sensitivity–specificity imbalance (e.g., LDS with lower specificity in the available study) (Table 2). Overall, these results indicate that high-ranked models by accuracy generally maintained strong sensitivity, while specificity differences were more modest and less consistently reported across studies.

**Table 2:** Summary of sensitivity and specificity across algorithms.

Algorithm	Studies reporting sensitivity	Mean sensitivity (%)	Sensitivity rank^a^	Studies reporting specificity	Mean specificity (%)	Specificity rank^a^
ResNet	1	94.0	1	1	93.0	1
DenseNet	2	91.5	2	2	85.5	5
LDS	1	89.0	3	1	52.0	8
SVM	5	86.8	4	5	86.6	3
MP	1	85.0	5	1	90.0	2
Bayes	2	83.0	6	2	86.0	4
CNN	2	80.0	7	2	82.0	7
ELM	1	69.0	8	1	84.0	6
FFR	0	–	–	0	–	–
NN	0	–	–	0	–	–
LR	0	–	–	0	–	–

a Ranks are assigned in descending order of the mean sensitivity or specificity (1 = highest). Algorithms with no reported values are not ranked.

### Publication bias

Visual inspection of the funnel plot suggested slight asymmetry, and Egger’s test indicated potential small-study effects (*p* = 0.029). Given the limited evidence base and heterogeneous study designs, this finding was interpreted cautiously; nevertheless, it raises the possibility that pooled advantages for certain models could be inflated by selective reporting and emphasizes the importance of publishing negative or null diagnostic modeling results.

## Discussion

Alzheimer’s disease (AD) poses a significant global health challenge, with projections indicating that by 2050, one in every 85 individuals worldwide will be afflicted by this condition (Eratne et al., 2018; Alzheimer’s Association, 2016; The Lancet, 2016; Aisen et al., 2017). Despite its prevalence, the etiology and pathogenesis of AD remain elusive, presenting a bottleneck in research efforts aimed at diagnosis and treatment. Recent evidence suggests that AD exhibits extensive heterogeneity across etiology, clinical presentation, pathological changes, and mechanisms (Zhang B. et al., 2021). Although memory impairment is a hallmark symptom, cognitive decline varies widely among individuals, encompassing deficits in memory, executive function, language, and spatial perception (Tan et al., 2014; Watanabe et al., 2019). However, the exact interplay of these pathogenic factors and their contribution to the disease’s progression remain incompletely understood. Moreover, recent advances in our understanding of AD have revealed its extensive heterogeneity across various domains, including etiology, clinical manifestation, pathological changes, and underlying mechanisms (Graff-Radford et al., 2021). While memory impairment stands as a primary symptom, the cognitive decline observed in AD patients encompasses a spectrum of deficits, ranging from memory loss to impairments in executive function, language, and spatial perception (Zhang et al., 2023). The complex manifestations of AD highlight the inadequacy of traditional diagnostic approaches, which often rely on subjective clinical assessments and lack the sensitivity to detect subtle changes in brain structure and function. In this regard, neuroimaging techniques, particularly magnetic resonance imaging (MRI), have emerged as invaluable tools for studying AD-related alterations in the brain (Bozzali et al., 2016; Almeida et al., 2024; Whitwell, 2010). However, traditional MRI-based analyses have primarily focused on predefined regions of interest, overlooking the intricate interplay between different brain regions (Dallaire-Théroux et al., 2017). To address this limitation, a paradigm shift toward network-based analyses has gained momentum in the field of AD research (Bailey and Hoy, 2021). By leveraging computational algorithms and functional connectivity analyses, researchers can delineate the complex interactions between brain regions and identify patterns of dysfunction associated with AD (Gherardini et al., 2023; Wang et al., 2022; Kincses et al., 2015).

In this systematic review and network meta-analysis, we synthesized comparative evidence across commonly used machine learning and deep learning approaches for MRI-based AD-related classification, with emphasis on methodological interpretation rather than repeating established background.

In the present systematic review and network meta-analysis, we compared machine learning and deep learning algorithms for MRI-based AD-related classification tasks and found that modern convolutional architectures generally achieved higher diagnostic performance than traditional classifiers. In summary, while the performance of each method varies across different studies, ResNet appears to be one of the most effective methods, followed closely by CNN and DenseNet, and ELM also demonstrated competitive performance in some comparisons. Bayes consistently performs poorly compared to other methods, while LR, MP, and SVM show comparable performance to it in many cases. To provide a more clinically informative evaluation beyond accuracy, we also considered sensitivity and specificity where available, recognizing that high sensitivity may occur at the expense of specificity depending on the algorithm and thresholding strategy. The confidence intervals provide valuable insights into the certainty of these accuracy estimates, helping researchers interpret the results with confidence. These findings highlight that accuracy-based ranking may not fully reflect clinically relevant trade-offs between false-negative and false-positive errors.

There are many models be built to make advancements confronting challenges in diagnosing Alzheimer’s disease (AD), leveraging various methodologies from computational techniques to advanced imaging technologies. One of the primary challenges highlighted is the absence of a standard diagnostic method for dementia, including AD, leading to difficulties in accessing effective treatment (Bloch and Friedrich, 2019; Bron et al., 2022). The World Alzheimer Report 2016 underscores the urgency, projecting a significant increase in the number of dementia cases by 2050 (Pettigrew et al., 2017). This sets the stage for exploring innovative approaches to early diagnosis. Several computational methods are proposed to address this challenge, focusing on utilizing deep learning techniques applied to brain MRI scans (Yao et al., 2023). These methods aim to enhance both segmentation and classification processes, crucial for accurate diagnosis (Li et al., 2022). The integration of Gaussian mixture model (GMM) and convolutional neural network (CNN) for segmentation, along with extreme gradient boosting (XGBoost) and support vector machine (SVM) for classification, demonstrates promising (Tuan et al., 2022; Liang et al., 2021). However, the discussion also acknowledges the computational costs and data limitations associated with traditional 3D CNNs in medical imaging analysis. To overcome these hurdles, researchers propose leveraging 2D CNNs on 3D MRI data, resulting in improved model performance with reduced training time (Sethi et al., 2022). Furthermore, the potential of multimodal neuroimaging fusion techniques, such as the cross-modal transformer generative adversarial network (CT-GAN), is highlighted. This approach effectively integrates functional and structural information from fMRI and DTI data, offering new insights into AD-related brain connectivity and improving prediction performance (Zuo et al., 2023). While these advances are encouraging, differences in preprocessing, feature engineering, and validation procedures can materially influence reported performance and complicate direct comparisons across studies. Beyond imaging techniques, the discussion extends to the application of nanotechnology and machine learning in AD theranosis. Nanoparticles, particularly superparamagnetic iron oxide nanoparticles (SPIONs), hold promise for both diagnosis and therapy, demonstrating potential in modulating mitophagy to mitigate AD-related neurodegeneration (Amiri et al., 2013; Xie et al., 2022). Moreover, the role of AI-driven diagnostics in analyzing MRI images for AD diagnosis is emphasized. Deep learning methodologies, such as pre-trained CNN models like ResNet50, offer automatic feature extraction, streamlining the diagnostic process and achieving high accuracy rates (Zhang and Liu, 2018; Chen et al., 2022). Consistent with this, moderate network heterogeneity (network *I*^2^ = 48%) indicates that study-level differences contribute meaningfully to variability in pooled estimates; subgroup comparisons suggest broadly stable algorithm rankings but potentially different effect magnitudes across tasks and modalities. In addition, potential small-study effects suggest that selective reporting may inflate apparent differences between algorithms, and further large-scale studies with transparent reporting and external validation are required.

Our study extends beyond imaging techniques to encompass the potential of nanotechnology and machine learning in AD theranosis, highlighting the promise of AI-driven diagnostics in streamlining the diagnostic process. Despite persistent challenges such as data heterogeneity and interpretability of AI-driven models, the collective efforts outlined in this discussion pave the way for transformative advancements in AD diagnosis and management. Through interdisciplinary collaboration and innovation, computational techniques and advanced imaging technologies hold the key to revolutionizing our understanding and approach to Alzheimer’s disease.

## Data Availability

The original contributions presented in the study are included in the article/supplementary material, further inquiries can be directed to the corresponding author.
